# Calcineurin A beta deficiency ameliorates HFD-induced hypothalamic astrocytosis in mice

**DOI:** 10.1186/s12974-018-1076-x

**Published:** 2018-02-08

**Authors:** Katrin Pfuhlmann, Sonja C. Schriever, Beata Legutko, Peter Baumann, Luke Harrison, Dhiraj G. Kabra, Emily Violette Baumgart, Matthias H. Tschöp, Cristina Garcia-Caceres, Paul T. Pfluger

**Affiliations:** 1 Research Unit NeuroBiology of Diabetes, Helmholtz Diabetes Center, Helmholtz Zentrum München, 85764 Neuherberg, Germany; 2 Institute for Diabetes and Obesity, Helmholtz Diabetes Center, Helmholtz Zentrum München, 85764 Neuherberg, Germany; 3 Division of Metabolic Diseases, Technische Universität München, 80333 Munich, Germany; 4 German Center for Diabetes Research (DZD), 85764 Neuherberg, Germany; 5 Institute for Clinical Biochemistry and Pathobiochemistry, German Diabetes Center. Heinrich Heine University, Leibniz Center for Diabetes Research, 40225 Düsseldorf, Germany

**Keywords:** Calcineurin, Ppp3cb, Obesity, Inflammation, Astrocytosis, Microgliosis, Hypothalamus

## Abstract

Astrocytosis is a reactive process involving cellular, molecular, and functional changes to facilitate neuronal survival, myelin preservation, blood brain barrier function and protective glial scar formation upon brain insult. The overall pro- or anti-inflammatory impact of reactive astrocytes appears to be driven in a context- and disease-driven manner by modulation of astrocytic Ca^2+^ homeostasis and activation of Ca^2+^/calmodulin-activated serine/threonine phosphatase calcineurin. Here, we aimed to assess whether calcineurin is dispensable for astrocytosis in the hypothalamus driven by prolonged high fat diet (HFD) feeding.

Global deletion of calcineurin A beta (gene name: Ppp3cb) led to a decrease of glial fibrillary acidic protein (GFAP)-positive cells in the ventromedial hypothalamus (VMH), dorsomedial hypothalamus (DMH), and arcuate nucleus (ARC) of mice exposed chronically to HFD. The concomitant decrease in Iba1-positive microglia in the VMH further suggests a modest impact of Ppp3cb deletion on microgliosis. Pharmacological inhibition of calcineurin activity by Fk506 had no impact on IBA1-positive microglia in hypothalami of mice acutely exposed to HFD for 1 week. However, Fk506-treated mice displayed a decrease in GFAP levels in the ARC. In vivo effects could not be replicated in cell culture, where calcineurin inhibition by Fk506 had no effect on astrocytic morphology, astrocytic cell death, GFAP, and vimentin protein levels or microglia numbers in primary hypothalamic astrocytes and microglia co-cultures. Further, adenoviral overexpression of calcineurin subunit Ppp3r1 in primary glia culture did not lead to an increase in GFAP fluorescence intensity.

Overall, our results point to a prominent role of calcineurin in mediating hypothalamic astrocytosis as response to acute and chronic HFD exposure. Moreover, discrepant findings in vivo and in cell culture indicate the necessity of studying astrocytes in their “natural” environment, i.e., preserving an intact hypothalamic microenvironment with neurons and non-neuronal cells in close proximity.

**Electronic supplementary material:**

The online version of this article (10.1186/s12974-018-1076-x) contains supplementary material, which is available to authorized users.

## Introduction

Astrocytes represent a population of specialized glial cells with important regulatory functions in the CNS. Astrocytes are located at the interface between neurons and blood vessels [1], where they facilitate the transfer of signaling molecules and nutrients between neurons and blood vessels [2]. Each astrocyte occupies a specific territory with its small finger-like processes that can interact with individual neighboring neurons, synapses [3] and influence synaptic transmission and plasticity [4]. Such astrocytic domains are dynamic with rapid changes depending on the surrounding microenviroment due to neuronal activity or metabolic changes.

Astrocytes can develop a reactive phenotype termed astrocytosis or astrogliosis characterized by a hypertrophy of the soma and cellular processes associated with an increase in GFAP and vimentin [5] as well as extracellular matrix molecules, growth factors, inflammatory cytokines, and oxidative stress markers [6]. Depending on the cellular and environmental context, the induction of astrocytosis is governed by various cellular signaling pathways. Astrocytosis occurs not only in brain injuries reflecting healing processes [7] and pathophysiologic conditions, but also in response to high fat diet feeding [8, 9]. HFD-induced astrocytosis is often found in the hypothalamus, a major center for the control of energy and glucose homeostasis, and associated with an increase in HFD-induced inflammation [10–12] and microglia activation [13–16]. How exactly HFD-induced astrocytosis is linked with hypothalamic inflammation and microglial activation remains elusive.

Crucial cellular signals in the induction of astrocytosis are alterations in Ca^2+^ homeostasis and the subsequent activation of Ca^2+^/calmodulin-activated serine-threonine phosphatase calcineurin (gene name protein: phosphatase 3, Ppp3). Calcineurin consists of two subunits, a 61 kD calmodulin-binding catalytic subunit A (Ppp3c) that is encoded by three genes: Ppp3ca, Ppp3cb or Ppp3cc, and a 19 kD Ca^2+^-binding regulatory subunit B (Ppp3r) that is encoded by the genes Ppp3r1 and Ppp3r2 [17]. Calcineurin activation was linked with anti-inflammatory IGF1 signaling in primary astrocytes [18]. Anti-inflammatory effects of activated calcineurin were further reported in vivo in a murine model of cortical brain injury, and in cell culture in primary astrocytes isolated from the cortex and hippocampus [19]. In contrast, calcineurin overexpression was reported to be sufficient to triggering pro-inflammatory astrocytosis in cultured hippocampal astrocytes [20].

We recently showed that mice with a global knockout of Ppp3cb were protected from high fat diet-induced obesity [21]. However, hypothalamic astrocytosis in response to chronic high fat diet exposure was not studied in these mice, and the contribution of calcineurin signaling to HFD-induced astrocytosis remains elusive. We here aimed to delineate whether calcineurin plays a role in the induction of hypothalamic astrocytosis induced by chronic HFD feeding. We specifically hypothesized that calcineurin ablation protects mice from HFD-induced hypothalamic astrocytosis. We further aimed to assess whether results observed in vivo can be reproduced in primary hypothalamic astrocyte cells after genetic or pharmacological manipulation of calcineurin activity.

## Methods

### Animals

Ppp3cb (calcineurin A beta) WT and KO mice [22–24] were generated as described previously [21]. C57BL/6J mice for the isolation of primary astrocytes were originally obtained from Janvier Lab (Saint-Berthevin Cedex, France) and were bred in our own facilities. Mice were maintained on a 12-h dark/light cycle and had free access to diet and water. Mice were exposed to high fat diet (Research Diets D03082706, New Brunswick, NJ, USA) containing 40% of calories from butter fat for 16 weeks. Mice were perfused and brains were collected for immunohistochemical stainings. Calcineurin inhibitor Fk506 (Enzo Life Sciences, #ALX-380-008-M005) at a dose of 1 mg/kg body weight (vehicle: 1% DMSO in PBS) was injected subcutaneously in 12-week-old male C57BL/6J mice at the onset of the dark phase. Fk506 was dissolved in pure DMSO, aliquots were frozen at − 20 °C and were diluted 1 to 100 with PBS every day before injections. All mice were fed chow diet before the experiment; at the start of the Fk506 injections, one cohort of 20 mice was exposed to HFD containing 58% kcal of calories from fat, while a second cohort of 12 mice continued to receive standard chow diet. All mice were sacrificed after a 2-h fast in the beginning of the light phase, i.e., 14 h after the final seventh treatment with Fk506 or vehicle. Hypothalami were either snap-frozen for qPCRs or excised after perfusion with 4% PFA for immunohistochemical stainings. All studies were approved by and performed according to the guidelines of the State of Bavaria, Germany.

### Body composition and blood glucose measurement

Nuclear magnetic resonance (NMR) technology (EchoMRI, Houston, TX, USA) was used to assess fat and lean mass. Tail blood glucose levels were measured using a handheld glucometer (Freestyle freedom Lite, Abbott Diabetes Care, Alameda, CA, USA).

### Primary glia culture

Hypothalami were extracted from C57BL/6J mice at postnatal days 1 to 4 and triturated in MEM. The suspension was centrifuged, and the cell pellet re-suspended in MEM containing 10% FBS. Cells were grown in T175 flasks for up to 14 days and afterwards seeded in 6- or 12-well plates for protein extraction, or 24-well plates or glass cover slips in 24-well plates for immunocytochemistry. Before seeding, microglia were partially removed by heavy taping.

For immunocytochemistry of Fk506-treated cells, 25,000 primary astrocytes were seeded on glass cover slips in a 24-well plate. Glass cover slips were pre-coated with 100 μM poly-L-lysine overnight the day before seeding and poly-L-lysine was washed away with PBS before the experiment. 225,000–350,000 cells were seeded in 6- or 12-well cell-bind-plates. 1 to 3 days after seeding, primary astrocytes were serum-fasted for 4 to 5 h with MEM in the absence of FBS. DMSO as control or calcineurin inhibitor Fk506 were added in indicated concentrations and 0.5 h later 10% FBS or 10% obese serum were added. 24, 48, or 72 h later cells were harvested for protein extraction or fixed with 4% PFA for immunocytochemistry. For RNA and protein extraction or immunocytochemistry of adenovirus-treated cells, 30,000 primary astrocytes were seeded on pre-coated poly-L-lysine glass cover slips in 24-well plates. Adenoviruses were applied to primary astrocytes in normal culture media (MEM, 10% FBS), viruses were washed from plates with PBS 12 h later and primary astrocytes were cultured for 72 h. 24 h before harvesting, media were refreshed with MEM containing 10% FBS or replaced by MEM supplemented with 10% obese serum. Cells were harvested for RNA or protein extraction, or fixed with 4% PFA for immunocytochemistry. Serum from diet-induced obese mice (body weights above 45 g) was collected by centrifugation of blood for 15 min at 2500 g and 4 °C.

### Immunohistochemistry

For immunohistochemical analyses, mice were perfused with PBS and a cold 4% solution of paraformaldehyde (PFA). Brains were incubated at 4 °C overnight in 4% PFA solution and afterwards incubated for 24–48 h in 30% sucrose in 0.1 M tris-buffered saline (TBS) at 4 °C. 30-μm-thick cryo-cuts were performed in a cryostat, and sections were stored in a solution containing glycerol, ethylene glycol, and sucrose dissolved in TBS. Stainings were performed with free-floating slices. Brain cuts were washed three times with TBS and were blocked for 2 h with a blocking buffer containing 0.25% gelatin and 0.5% Triton X 100 in 1× TBS. For DAB staining in Ppp3cb KO vs. WT mice, GFAP (1/1000, anti-rabbit, Dako, #Z0334) and Iba1 (1/500, anti-rabbit, Synaptic System, #234003) primary antibodies were diluted in blocking buffer and incubated overnight at 4 °C. Secondary anti-rabbit biotinylated antibody (1/1000, anti-rabbit IgG (H + L), Vector Laboratories, #BA-1000) was added for 1 h at room temperature after several washing steps in TBS. Brains were incubated in ABC reagent for 1 h at room temperature followed by stainings with diamino-benzidine and H_2_O_2_. Brain cuts were dehydrated with increasing concentrations of ethanol and were delipidated using xylene. Fluorescence stainings in mice were conducted with primary antibodies for GFAP (1/1000, anti-mouse, Sigma-Aldrich, #G3893), Iba1 (1/500, anti-rabbit, Synaptic System, #234003), or Cd169 (1/200, anti-mouse, Biorad, MCA884GA). Secondary antibodies used were Alexa Fluor 488 goat anti-rabbit IgG (H + L; 1/1000, Life Technologies, #A11008) and Alexa Fluor 568 donkey anti-mouse IgG (H + L; 1/1000, Life Technologies, #A10037). Sections were mounted on glass slides using mounting media (Permount, Fisher Scientific, #SP15-500). Images were captured by a BZ-9000 microscope (Keyence Corporation Itasca, IL, USA) or a Leica TCS SP8 microscope (Leica Microsystems, Wetzlar, Germany).

### Quantification of GFAP or Iba1 positive cells in the hypothalamus

For quantification of GFAP- or Iba1-positive cells in WT vs. Ppp3cb KO mice in the ARC, rectangles of equal size (0.0031 cm^2^) per picture were quantified for positively stained cells. Moreover, in the respective rectangles, astrocytes were assessed for the number and length of primary projections. The areas of the DMH and VMH were assigned for each picture individually by using the mouse brain atlas. Positive nuclei in the assigned areas were counted manually and normalized for the area surface. Primary projections of 15 ARC cells/picture, 10 DMH cells/picture, and 2-5 VMH cells/picture were counted. The length of the primary projections in the ARC was measured in 15 cells/picture. A total of six animals per group were used, and a minimum of four technical replicates (brain hemispheres and slices) were analyzed per animal, if not indicated otherwise in the figure legends. For quantification of GFAP and IBA1 fluorescence intensities in Fk506 vs. vehicle-injected WT mice, the ARC, VMH, and DMH were assigned for 2–4 hemispheres per mouse brain. Total fluorescence intensity was measured using ImageJ software, and mean hemisphere values per mouse were used for statistical analyses.

### Immunocytochemistry

PFA was washed away from glass cover slips with PBS. Slides were incubated with blocking buffer containing 5% goat serum and 0.3% Trition X-100 for 1 h. GFAP (1/1000, anti-rabbit, Dako, #Z0334) primary antibody was added overnight in blocking solution. Three PBS washing steps were performed and Alexa Fluor 488 goat anti-rabbit IgG (H + L) secondary antibody (1/1000, Life Technologies, #A11008) was added for 1 h in blocking solution. Cover slips were washed again with PBS and mounted afterwards using mounting media containing DAPI (Vectashild, Vector, #H-1200). Images were captured by a BZ-9000 microscope (Keyence Corporation Itasca, IL, USA). Total fluorescence intensity was determined with ImageJ 1.47v software.

### Cell viability assay

Cell viability was measured with MTT and Alamar (Thermo Fisher Scientific, DAL1025) assays. For the MTT assay, 0.16 × 10^5^ cells were seeded per well of a 96-well plate, with 7 replicates per group. 3 days later, astrocytes were fasted with MEM (5.56 mM glucose) for 4 to 5 h, Fk506 was added in different concentrations (with 0.1% DMSO) and half an hour later 10% FBS was added to the wells. 1 day later, the MTT assay was performed. For the assay, the media in the plates were changed to 100 μl fresh MEM media (10% FBS). Per condition, one dead control (3 μl of 10% Triton) was used and 10% MTT (10 μl) was added to each well. Plates were placed in a normal cell culture incubator for 1 h and afterwards media were removed and 100 μl solubilization buffer (containing isopropanol, triton X-100, and HCl) was added. After 10 min, absorption was measured at wavelengths 570 and 650 nm. For calculations, the 650 nm value was subtracted from the 570 nm value and astrocytic survival was calculated in % of DMSO treated cells. The Alamar assay was performed according to the manufacturer’s protocol. Absorption was measured at wavelength 570 and 600 nm, and the 570 nm value was normalized to the 600 nm value. Astrocytic survival was calculated in % of DMSO-treated cells.

### Western blotting and densitometric analyses

Proteins were extracted using RIPA buffer containing protease and phosphatase inhibitor cocktail (Thermo Fisher Scientific Inc., Rockford, IL, USA) and 1 mM phenyl-methane-sulfonyl fluorid (PMSF). Proteins were transferred from the gel to the nitrocellulose membranes using a Trans Blot Turbo transfer apparatus (Biorad, Hercules, CA, USA). Primary antibodies for GFAP (1/4000, anti-mouse, Sigma-Aldrich, #G3893), NFATc4 (1/1000, anti-rabbit, Santa Cruz Biotechnology, #sc-13,036), β-Actin (1/10000, anti-rabbit, Cell Signaling Technology, #4970), vimentin (1/2000, anti-mouse, Sigma, #V5255), or CB1 (1/2000, anti-rabbit, Millipore, #07-069) were added to the membranes in 5% BSA TBS-T and were incubated overnight. Membranes were subjected to ECL (Biorad, Hercules, California, USA) detection on a LiCor Odyseey instrument (Lincoln, NE, USA).

### RNA extraction, cDNA synthesis, and qPCR

RNA of tissues and cells was isolated using a commercially available kit (Macherey-Nagel, Düren, Germany). Equal amounts of RNA were transcribed to cDNA using the QuantiTect Reverse Transcription kit (Qiagen, Hilden, Germany). Gene expression was analyzed using TaqMan probes (Thermo Fischer Scientific, Inc., Rockford, IL USA) and TaqMan mastermix (Applied Biosystem, Carlsbad, CA, USA). Gene expression was evaluated using the delta-delta Ct method and *Hprt* was used as the housekeeping gene. The following TaqMan probes were used: Gfap (Mm01253033_m1), Iba1 (Mm00479862_g1), Il1b (Mm00434228_m1), Il6 (Mm01253033), Tnf (Mm00443258_m1), Cd169 (Mm00488332_m1), Ppp3ca (Mm01317678_m1), Ppp3cb (Mm00920265_m1), Ppp3r1 (Mm01187904_m1), Ppp3r2 (Mm01349242_g1), Nfatc2 (Mm01240677_m1), Nfatc3 (Mm01249200_m1), Cox2 (Mm03294838_g1), Rcan1 (Mm01213406_m1). In general, we found low expression levels for hypothalamic inflammatory markers, with mean Ct values of 33 and values ranging from 31 to 35.

## Results

### Global loss of Ppp3cb prevents an increase of GFAP-positive astrocytes in the hypothalamus in response to HFD feeding

**Fig. 1:**
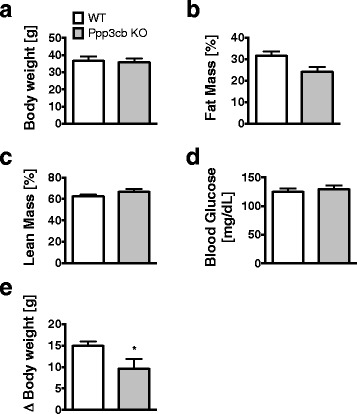
Body weight, body composition, and blood glucose of WT and Pppcb KO mice. Subgroups of WT and Ppp3cb KO mice matched for body weight (**a**), fat mass (**b**), lean mass (**c**), and blood glucose levels after 5 h of fasting (**d**) were selected from a larger cohort of WT and Ppp3cb KO mice that were exposed to HFD for 16 weeks. The selected Ppp3cb KO mice nevertheless showed a decreased body weight gain over the period of HFD feeding when compared to their selected WT controls (e). *n* = 6. Means ± SEM. Student’s *t* test: *p < 0.05

We recently showed that mice lacking the calcineurin subunit A beta (gene name: Ppp3cb) were protected from diet-induced obesity (DIO) [21]. Here, we aimed to assess whether calcineurin ablation affects the activation of astrocytes in mice chronically exposed to HFD. We first selected body weight (BW) matched Ppp3cb WT and KO mice from a large cohort of HFD-fed mice to account for the impact of body weight as covariate for HFD-induced astrocytosis (Fig. 1a). The sub-selection of BW-matched Ppp3cb WT and KO mice was associated with comparable fat (Fig. 1b) and lean mass (Fig. 1c) as well as blood glucose levels (Fig. 1d) between genotypes. HFD-fed Ppp3cb KO mice nevertheless displayed partial protection from DIO compared to WT mice, which was based on initially elevated body weights paired with lower body weight gain in the 16 weeks of HFD feeding (Fig. 1e).

**Fig. 2:**
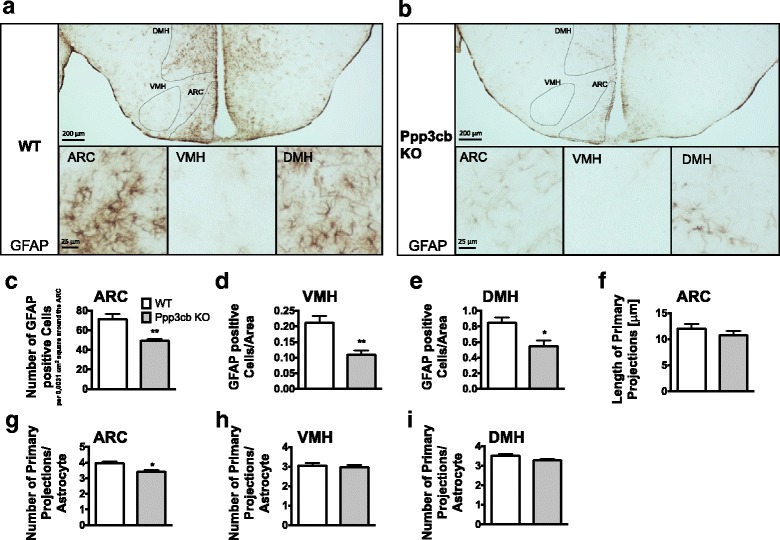
Ppp3cb deficiency is associated with a decrease in GFAP-positive cells in the ARC, DMH, and VMH of HFD-fed mice. Immunohistochemical stainings for GFAP in the arcuate nucleus (ARC), dorsomedial hypothalamus (DMH), and ventromedial hypothalamus (VMH) of HFD-fed Ppp3cb WT and KO mice. Representative images of the mediobasal hypothalamus (**a**, **b**, upper pictures) and of astrocytes in the ARC, VMH, DMH (**a**, **b**, lower pictures) of HFD-fed Ppp3cb WT, and KO mice. Numbers of GFAP-positive cells in the ARC (**c**), VMH (**d**), and DMH (**e**). Length of primary projections in the ARC of Ppp3cb WT and KO mice (**f**). Numbers of primary projection per astrocyte in the ARC (**g**), VMH (**h**), and DMH (**i**). Means ± SEM; *n* = 6 biological replicates per genotype (four pictures per biological replicate were quantified) except for (**d**), where only five KO brains could be used and (**f**) where length could be measured only in four KO brains; unpaired two-tailed Student’s *t* test. *p < 0.05, **p < 0.01

Next, we quantified the number of GFAP-expressing astrocytes in metabolically relevant hypothalamic nuclei of HFD-fed Ppp3cb WT and KO mice by using immunohistochemical stainings for GFAP (Fig. 2a, b). We found fewer GFAP-positive cells in the arcuate nucleus of the hypothalamus (ARC), ventromedial nucleus of the hypothalamus (VMH) and in the dorsomedial nucleus of the hypothalamus (DMH) of HFD-fed Ppp3cb KO mice compared to WT controls (Fig. 2c–e). The length of primary projections in the ARC remained unchanged between genotypes (Fig. 2f), but the overall number of primary projections in ARC astrocytes was decreased in Ppp3cb KOs (Fig. 2g). No difference in the number of primary projections was observed in the VMH (Fig. 2h), but a strong tendency towards a decrease in the number of primary projections was observed in the DMH (*p* = 0.057; Fig. 2i). Overall, these findings suggest that Ppp3cb deficiency can prevent HFD-induced increases in the number of GFAP-expressing hypothalamic astrocytes without having major effects on the length and number of primary projections.

### Ppp3cb deficiency decreases the number of microglia in the VMH of HFD-fed mice

**Fig. 3:**
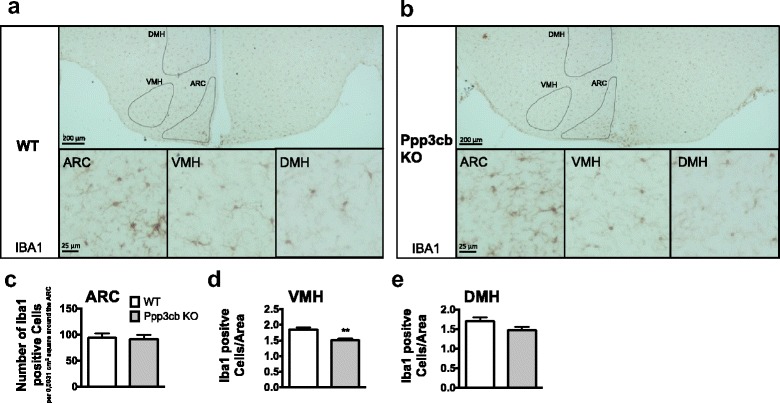
Ppp3cb deficiency decreases the number of Iba1 positive cells in the VMH of HFD-fed mice. Immunohistochemical staining for Iba1 in the ARC, DMH, and VMH of Ppp3cb WT (**a**) and KO (**b**) mice and quantitative analysis of Iba1-positive microglia (**c**–**e**) in the ARC (**c**), VMH (**d**), and DMH (**e**). Means ± SEM; *n* = 6 WT and five KO mice (**c**), *n* = 6 mice per genotype (**d**, **e**); *n* = 4 pictures per biological replicate were quantified. Unpaired two-tailed Student’s *t* test. **p < 0.01

Changes in the number of GFAP-positive astrocytes are often associated with changes in the number of microglia. HFD feeding was shown to simultaneously increase the expression of GFAP and microglia markers [8]. A recent report further suggested microglia activation as crucial driver for the activation of a subset of astrocytes [14]. We therefore quantified the number of cells positive for microglia marker Iba1 in the hypothalamus of HFD-fed Ppp3cb WT and KO mice (Fig. 3a, b). HFD-fed Ppp3cb KO mice did not show differences in the number of Iba1-positive microglia in the ARC (Fig. 3c) or in the DMH (Fig. 3e). However, Iba1-positive cells were decreased in the VMH of HFD-fed Ppp3cb KO mice compared to WT controls (Fig. 3d).

To detect whether changes in GFAP and IBA1 levels are associated with monocyte infiltration into the hypothalamus, we conducted immunohistochemical stainings for Cd169 as a marker for blood-derived monocytes [16, 25, 26]. In the hypothalamus of Ppp3cb KO and WT mice, we were unable to detect Cd169+ Iba1+ cells. In the adjacent median eminence (ME), limited numbers of Cd169+ Iba1+ double-positive cells were found in both Ppp3cb KO and WT mice (Additional file 1: Figure S1a). Moreover, Cd169 staining was confirmed by detecting high monocyte numbers in the spleen and no signal in spleens stained only with secondary antibody (Additional file 1: Figure S1b).

Taken together, these data show lower numbers of IBA1-positive microglia and GFAP-expressing astrocytes in the VMH but disparate HFD-induced activation of GFAP vs. IBA1 in the ARC and DMH of HFD-fed Ppp3cb KO mice compared to WT controls.

### Pharmacological calcineurin inhibition decreases GFAP fluorescence intensity in the ARC of mice acutely exposed to HFD

**Fig. 4:**
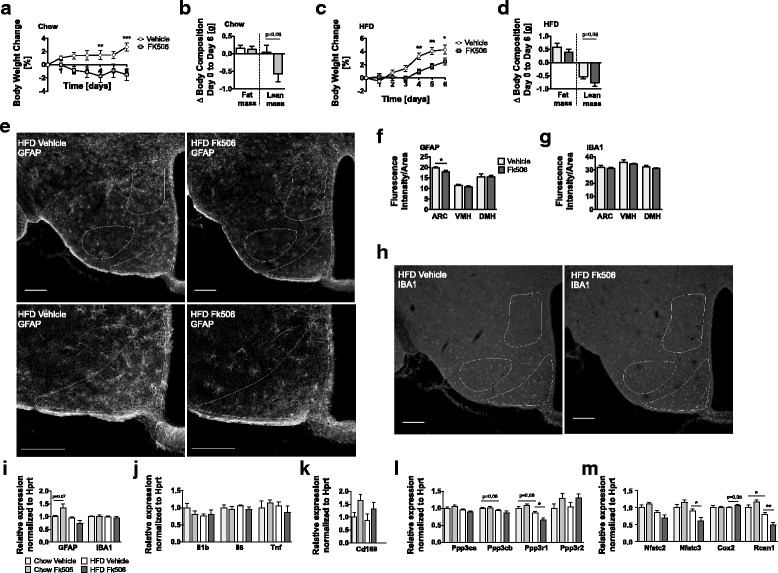
Pharmacological inhibition of calcineurin decreases GFAP fluorescence intensity in the ARC of mice acutely exposed to HFD. WT mice on chow (**a**, **b**) and HFD (**c**, **d**) were treated with 1 mg/kg/day of calcineurin inhibitor Fk506 for 1 week; HFD feeding was started in parallel. Fk506 treatment resulted in a significant reduction in body weight in mice on chow (**a**) and HFD (**c**), while no significant changes in body composition on chow (**b**) and HFD (**d**) were detected. GFAP (**e**, **f**) and IBA1 (**g**, **h**) staining in the ARC, VMH, and DMH of vehicle and Fk506-treated WT mice on HFD (scale 200 μm) revealed a decrease in GFAP fluorescence intensity in the ARC (**e**, **f**) Real-time qPCRs of mice on chow and HFD (**i**–**m**) were performed for Gfap and Iba1 (**i**), inflammatory cytokines (**j**), Cd169 as marker for monocyte infiltration (**k**), calcineurin subunits (**l**), and calcineurin downstream targets (**m**). Means ± SEM; *n* = 6 biological replicates (**a**, **b**); *n* = 10 biological replicates, except for lean mass vehicle, where one value was identified as a significant outlier (**c**, **d**); *n* = 4 biological replicates (**f**, **g**); *n* = 6 biological replicates, except for Gfap chow vehicle, where one value was identified as a significant outlier (**i**–**m**)

We next aimed to assess whether pharmacological inhibition of calcineurin by Fk506 (1 mg/kg/day) can affect astrocytosis and microgliosis in C57BL/6J mice acutely exposed to HFD (58% kcal from fat). Additional groups of vehicle- or Fk506-treated mice were fed chow diet. In line with our previous report [21], Fk506 treatment in both chow and HFD fed mice led to a significant decrease of body weight and non-significant trend for decreased lean mass, compared to vehicle controls (Fig. 4a–d). GFAP fluorescence intensities were reduced in the ARC of Fk506-injected mice exposed to HFD, whereas GFAP fluorescence intensities remained unchanged in the VMH and DMH (Fig. 4e, f). Staining for Iba1 suggests similar numbers of microglia in HFD-fed mice injected with or without Fk506 in the ARC, VMH, and DMH. Gene expression levels of Gfap and Iba1 in the hypothalamus were similar for all treatment groups and diets (Fig. 4i). Similarly, inflammatory markers Il1b, Il6 and Tnf, and a marker for monocyte infiltration, Cd169, were unaltered between groups (Fig. 4j, k). Downregulation of calcineurin subunit Ppp3r1 (Fig. 4l) and calcineurin downstream targets Nfatc3 and Rcan1 (Fig. 4m) in response to Fk506 treatment in HFD-fed mice indicates impaired calcineurin activity.

### Unperturbed astrocyte morphology or GFAP gene expression in primary astrocytes treated with serum from obese mice and/or calcineurin inhibitor FK506*.*

Next, we aimed to translate our in vivo findings into a cell culture system, thereby delineating the mechanisms responsible for decreased HFD-induced Gfap and Iba1 activation in HFD-fed Ppp3cb KO mice. We specifically aimed to evaluate the impact of blood-borne metabolic factors derived from HFD feeding on astrocyte reactivity and Gfap expression, and aimed to assess whether this could be blocked by calcineurin inhibitor Fk506. Glial cells were isolated from the hypothalamus of male pups and were exposed to serum from DIO mice to mimic the conditions of an obesogenic environment. Moreover, we aimed to assess the impact of blood-borne metabolic factors derived from serum of DIO mice in the presence and absence of Fk506.

We first confirmed the effectiveness of Fk506 to inhibit calcineurin by showing a reduction in the hyperphosphorylation of NFAT (Additional file 2: Figure S2a). Likewise, we observed that Fk506 in concentrations ranging from 1 up to 30 μM had no effect on astrocytic cell survival (Additional file 2: Figure 2b). Next, we analyzed the morphology and protein levels of different astrocyte markers in primary hypothalamic cultures. Astrocytes showed changes in the morphology towards a more reactive phenotype, characterized by a distinct prolongation of astrocytic projections in response to DIO serum compared to astrocytes treated with FBS serum (Additional file 2: Figure S2c–e, left pictures). However, the addition of 2 or 5 μM of Fk506—regardless of whether they were treated with or without DIO serum—neither had an impact on protein levels of GFAP or vimentin in a western blot (Additional file 2: Figure S2a) nor on the morphology of primary astrocytes (Additional file 2: Figure S2c–d, middle and right pictures). The lack of effect of Fk506 on the morphology of astrocytes and GFAP protein levels was corroborated by immunohistochemical stainings for GFAP (5 or 10 μM Fk506; Additional file 2: Figure S2f), which revealed unchanged GFAP fluorescence intensities upon Fk506 treatment (Additional file 2: Figure S2g).

Interestingly, the addition of obese serum to primary astrocytes seemed to increase the number of microglia that were co-isolated with primary astrocytes. This increase in microglia number appeared 24 h after the addition of obese serum (Additional file 2: Figure S2c, left pictures) and became more apparent 48 h after the addition of obese serum (48 h: Additional file 2: Figure S2d, left pictures; 72 h: Additional file 2: Figure S2e, left pictures).

### Adenoviral overexpression of calcineurin subunit B1 in primary glia cells does not affect GFAP protein levels

Last, we aimed to assess whether adenoviral overexpression of calcineurin subunit B1 (gene name: Ppp3r1) can modulate GFAP protein levels in primary glia cells. Initially, overexpression of calcineurin B1 was confirmed by western blotting, which revealed increased protein levels compared to beta-galactosidase overexpressing control cells (Additional file 3: Figure S3a). Increased levels of calcineurin B1 were also detected by quantitative PCR (Additional file 3: Figure S3b). However, similar fluorescence intensities in glial cultures stained with anti-GFAP antibody suggest similar protein levels in all groups. Notably, a trend for increased GFAP staining intensities in both beta-galactosidase and calcineurin B1 overexpressing cells suggests modest effects of the adenovirus that are independent of the target gene (Additional file 3: Figure S3c). Overall, our data indicate that primary astrocyte cultures are not a suitable tool to study astrocytosis under obesogenic conditions in the context of calcineurin inhibition.

## Discussion

We show here for the first time that the genetic and pharmacological inhibition of calcineurin in mice protects from HFD-induced GFAP activation in hypothalamic nuclei, suggesting a prominent role for calcineurin in HFD-induced hypothalamic astrocytosis. Our findings extend a number of reports on calcineurin as mediator of astrocytosis in various models of brain insult [19, 20, 27–29]. Accordingly, our findings warrant further studies on the physiological role of astrocytic calcineurin in the development of metabolic dysfunction.

Astrocytosis as a response to chronic exposure of mice and humans to an obesogenic diet has been reported by others and us [2, 8, 30]. However, the role of HFD-induced astrocytosis for astrocyte function and survival, surrounding nerve tissue and the hypothalamic control of metabolism remains largely unknown. Future studies on the impact of HFD-induced astrocytosis for systemic metabolism are thus warranted. Such studies should entail research on the potential physiological impact of astrocyte-specific calcineurin ablation in mice exposed to a normal chow diet or obesogenic environment.

Calcineurin inhibition in astrocytes may serve as crucial step in protecting nerve tissue from traumatic brain insults. Murine models of aging and Alzheimer’s disease displayed high calcineurin immunoreactivity in activated astrocytes located in the hippocampus and neocortex, but undetectable immunoreactivity in non-activated normal astrocytes [20]. Proteolytic activation of calcineurin in activated astrocytes exacerbated neuronal dysfunction during neurodegenerative disease and injury [31]. Regulation of calcineurin by insulin-like growth factor I and interaction with the transcription factor FOXO3 were recently identified as prime drivers for inflammation in Alzheimer’s disease [19, 32], and blockade of calcineurin-FOXO binding decreased neuropathologies in murine models of Alzheimer’s disease [33]. Virally mediated inhibition of calcineurin signaling in astrocytes was shown to normalize hippocampal synaptic function and plasticity in rats undergoing traumatic brain injury [34]. Notably, such neuroprotective functions of astrocytic calcineurin inhibition were found despite the presence of GFAP activation and reactive gliosis [34].

A recent report demonstrated that mice with global Ppp3cb deficiency were unable to cope with brain injury, displaying a significant increase in cerebral damage due to the lack of unfolded protein response [35]. Astrocyte size and GFAP expression were decreased in brains of Ppp3cb KO mice following photothrombotic stroke or traumatic brain injury. Mechanistically, calcineurin A beta was directly linked with non-enzymatic activation of PERK-eIF2α signaling and the unfolded protein response, which facilitated astrocytosis as a function of increased astrocyte survival and protection from cerebral damage [35]. These data may imply that astrocytic Ppp3cb ablation could exert beneficial effects on metabolic homeostasis.

Previous cell culture studies linked astrocytic calcineurin with free-radical production and cellular damage in the presence of high calcium [36]. Inhibition of calcineurin by FK506 protected cultured astrocytes from Ca^2+^ paradox-like injury [36] and inhibited astrocyte proliferation and apoptosis [37], a process potentially involving inhibition of arachidonic acid release by cytosolic phospholipase A2 [38], increased MAP kinase Erk1/2, and Akt signaling [39] and/or the prevention of glutamate toxicity [40]. In vitro, adenoviral overexpression of calcineurin was linked with morphologic changes including thickening of somata and processes, and with gene expression profiles reminiscent of astrocytosis [20]. Adenoviral inhibition of astrocytic calcineurin activity reduced neuroinflammatory IL-1 signaling and neuronal death in co-cultures of neurons and glia cells [29]. We observed an increase in GFAP fluorescence intensity after adenoviral overexpression of calcineurin B1; however, these were also observed in beta-galactosidase overexpressing controls, indicating off-target effects driven by the adenoviral infection.

Overall, our own data indicate that cell culture experiments in isolated primary hypothalamic astrocytes are of limited value for addressing the role of calcineurin for HFD-induced astrocytosis or metabolic dysfunction. We were unable to corroborate the protective role of calcineurin A beta from HFD-induced astrocytosis observed in vivo in Ppp3cb KO or Fk506-treated C57Bl/6J mice. Rather, we found unchanged GFAP protein levels and unchanged general GFAP fluorescence intensity upon either calcineurin inhibition by Fk506 treatment or genetic overexpression of calcineurin subunit B1 in cell culture in primary astrocytes. After treating primary astrocytes with obese serum, we observed unchanged GFAP expression but a shift towards a more reactive morphology, which could not be blocked by calcineurin inhibition. Similarly and consistent with previous reports on increased Iba1-positive microglia in the ARC of HFD-fed mice [8, 13], we found increased microglia numbers in primary cultures treated with serum obtained from obese mice, regardless of the presence or absence of calcineurin inhibitor FK506. Calcineurin inhibition in these cells was confirmed by assessing NFAT phosphorylation via western blotting. Hypothalamic astrocytes in cell culture appear to behave differently compared to hypothalamic astrocytes in vivo. They may require close proximity to a network of other cell types, including neurons or non-neuronal cells. Differential astrocytic phenotypes in vitro may further result from subtle but distinct changes in culture conditions such as serum and cell confluence, which were shown to exert differential effects on astrocytic calcineurin-NFAT signaling [41]. Moreover, hypothalamic astrocytes, or subpopulations thereof, may differ greatly from astrocytes isolated from other brain regions. Putative differences in the function of calcineurin in distinct astrocytic subpopulations further complicate comparisons between studies, highlighting the plurality of astrocyte biology and function.

In summary, our results propose a novel role for calcineurin in HFD-induced hypothalamic astrocytosis. Murine models of calcineurin inhibition displayed decreased GFAP protein levels in the ARC, DMH, and VMH after chronic HFD exposure, and decreased GFAP levels in the ARC after an acute exposure to 1 week of HFD. The number of reactive microglia in the ARC and DMH, regardless of the duration of HFD exposure, was not affected by calcineurin loss-of-function. However, after chronic HFD exposure, we observed a decrease in the number of reactive microglia in the VMH. To date, the significance of these findings remains to be tested, and both an exacerbation and protection from metabolic diseases appear possible. Functional data should include a thorough metabolic characterization of mice with calcineurin-loss-of-function specifically in astrocytes. Ideally, such studies should further entail specific ablation of calcineurin activity from subpopulations of astrocytes, e.g., in the hypothalamus to help delineating the complexity and heterogeneity of astrocyte activation during injury and disease.

## Additional files

Additional file 1: Figure S1. Cd169-positive monocytes are present in the median eminence (ME) but not in the hypothalamus of HFD-fed Ppp3cb and WT mice. Immunohistochemical stainings for Cd169 revealed the absence of monocytes from the hypothalamus (a, upper pictures), and limited numbers of monocytes in the ME of Ppp3cb KO and WT mice. Cd169 positive cells in the ME were also Iba1 positive (a, middle and lower pictures). Cd169 stainings in the spleen of a WT mouse in the presence (left picture) or absence of Cd169 primary antibody (b). Scale bar 50 μm. (PDF 6623 kb)

Additional file 2: Figure S2. Effects of calcineurin inhibitor Fk506 and serum from obese mice on GFAP and vimentin levels, cell survival, and glial morphology in primary glial cultures. Representative western blot of GFAP and vimentin protein levels (a) after treatment of the primary glia cultures with FBS, obese serum, and/or Fk506 (2 or 5 μM); calcineurin inhibition was corroborated by revealing an Fk506-mediated decrease in NFATc4 protein levels; β-actin was used as housekeeping protein. Cell survival assay (b) showing cells treated with increasing concentrations of Fk506 for 48 h. Representative light microcopy images of primary astrocytes and co-isolated microglia treated with FBS or obese serum, and 2 or 5 μM Fk506 for 24 h (c), 48 h (d), and 72 h (e), respectively. Scale bar 100 μm. Representative pictures of immunocytochemical stainings (f) and quantification of fluorescence intensities to GFAP (green dye, g) in untreated, 5 μM Fk506 and 10 μM Fk506-treated primary astrocytes. Scale bar 100 μm. Means ± SEM; *n* = 2 biological replicates; calcineurin inhibition was confirmed once with NFATc4 (a); *n* = 3 biological replicates (b); representative pictures (c, d, e); quantification of GFAP staining was done in 10–11 pictures/treatment group (f, g). (PDF 817 kb)

Additional file 3: Figure S3. Unchanged GFAP fluorescence intensity after adenoviral overexpression of calcineurin subunit B1 (CB1). (a–c) Primary glia cultures were treated for 72 h with a control adenovirus overexpressing beta-galactosidase (Beta gal) or an adenovirus overexpressing CB1. Overexpression of CB1 increased CB1 protein levels (a) and mRNA levels (b), but had no effect on total GFAP fluorescence intensities compared to beta gal overexpressing cells (c). Beta gal: conc. 1: 38 μl/24-well, conc. 2: 75 μl/24-well; CB1: conc. 1: 75 μl/24-well, conc. 2: 150 μl/24-well. 1 (a, b) and 4–5 technical replicates from glia cells isolated from a pool of four male pups; means ± SEM (c). (PDF 147 kb)

## Abbreviations

Akt: Protein kinase B
ARC: Arcuate nucleus
BW: Body weight
CNS: Central nervous system
DAPI: (4′,6-diamidino-2-phenylindole)
DIO: Diet-induced obesity
DMH: Dorsomedial hypothalamus
DMSO: Dimethyl sulfoxide
eIF2: Eukaryotic initiation factor 2
Erk1/2: Extracellular signal-regulated kinase 1/2
FBS: Fetal bovine serum
FOXO3: Forkhead box O3
GFAP: Glial fibrillary acidic protein
HFD: High fat diet
Iba1: Ionized calcium-binding adapter molecule 1
IgG: Immunoglobulin G
MEM: Minimum essential medium
MTT: 3-(4,5-dimethylthiazol-2-yl)-2,5-diphenyltetrazolium bromide
NFAT: Nuclear factor of activated T-cells
NMR: Nuclear magnetic resonance
PERK: Protein kinase RNA-like endoplasmic reticulum kinase
PFA: Paraformaldehyde
PMSF: Phenyl-methane-sulfonyl fluorid
SEM: Standard error of the mean
Stat3: Signal transducer and activator of transcription 3
TBS: Tris-buffered saline
VMH: Ventromedial hypothalamus
