# Supplementary figures and images for: Calcineurin A beta deficiency ameliorates HFD-induced hypothalamic astrocytosis in mice
Source: J Neuroinflammation. 2018 Feb 8;15:35. doi: 10.1186/s12974-018-1076-x (PMC5806488; doi:10.1186/s12974-018-1076-x)

**a****WT**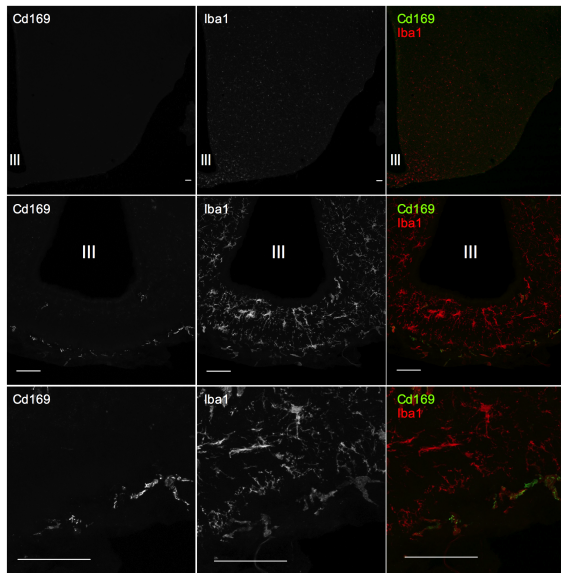**KO**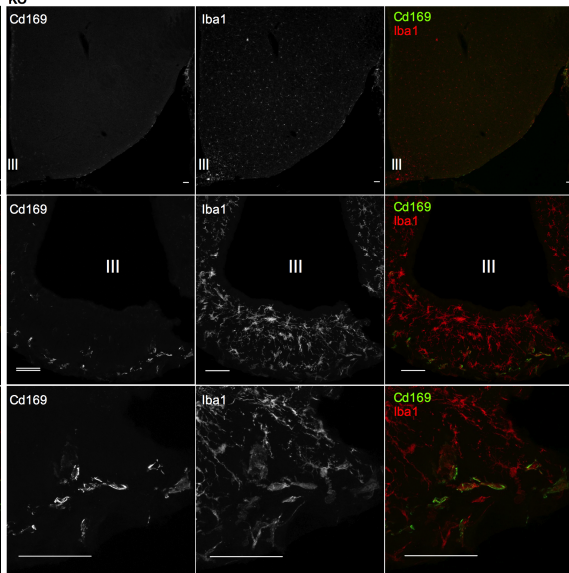**b****Spleen WT**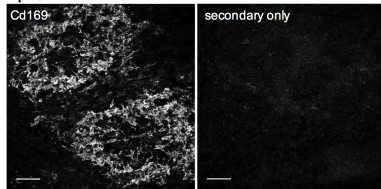

Supplement: Supplementary file 1 — Cd169-positive monocytes are present in the median eminence (ME) but not in the hypothalamus of HFD-fed Ppp3cb and WT mice. Immunohistochemical stainings for Cd169 revealed the absence of monocytes from the hypothalamus (a, upper pictures), and limited numbers of monocytes in the ME of Ppp3cb KO and WT mice. Cd169 positive cells in the ME were also Iba1 positive (a, middle and lower pictures). Cd169 stainings in the spleen of a WT mouse in the presence (left picture) or absence of Cd169 primary antibody (b). Scale bar 50 μm. (PDF 6623 kb) [file 12974_2018_1076_MOESM1_ESM.pdf]

**a**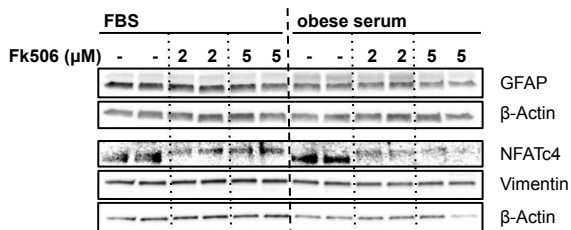**b**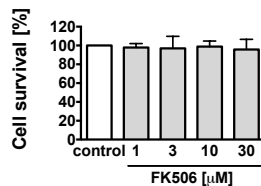**c****24h**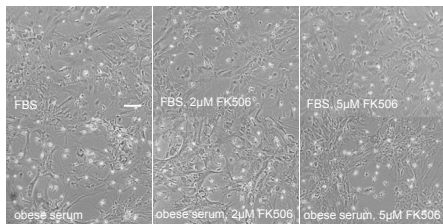**f**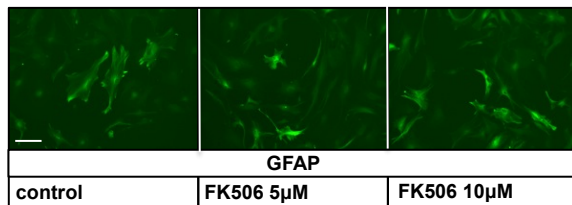**d****48h**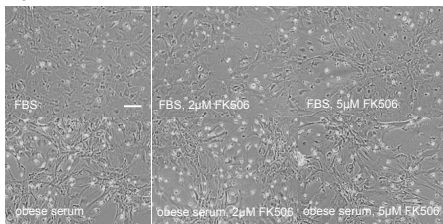**g**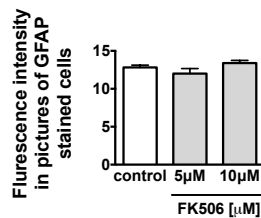**e****72h**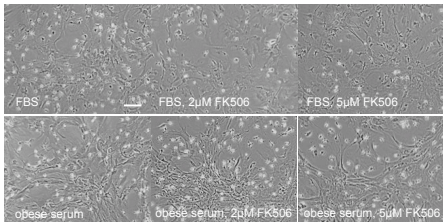

Supplement: Supplementary file 2 — Effects of calcineurin inhibitor Fk506 and serum from obese mice on GFAP and vimentin levels, cell survival, and glial morphology in primary glial cultures. Representative western blot of GFAP and vimentin protein levels (a) after treatment of the primary glia cultures with FBS, obese serum, and/or Fk506 (2 or 5 μM); calcineurin inhibition was corroborated by revealing an Fk506-mediated decrease in NFATc4 protein levels; β-actin was used as housekeeping protein. Cell survival assay (b) showing cells treated with increasing concentrations of Fk506 for 48 h. Representative light microcopy images of primary astrocytes and co-isolated microglia treated with FBS or obese serum, and 2 or 5 μM Fk506 for 24 h (c), 48 h (d), and 72 h (e), respectively. Scale bar 100 μm. Representative pictures of immunocytochemical stainings (f) and quantification of fluorescence intensities to GFAP (green dye, g) in untreated, 5 μM Fk506 and 10 μM Fk506-treated primary astrocytes. Scale bar 100 μm. Means ± SEM; n = 2 biological replicates; calcineurin inhibition was confirmed once with NFATc4 (a); n = 3 biological replicates (b); representative pictures (c, d, e); quantification of GFAP staining was done in 10–11 pictures/treatment group (f, g). (PDF 817 kb) [file 12974_2018_1076_MOESM2_ESM.pdf]

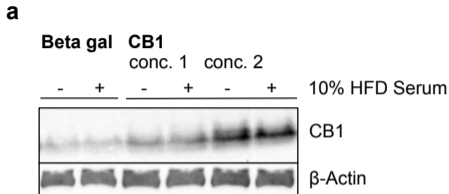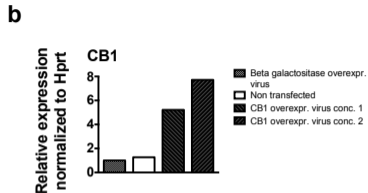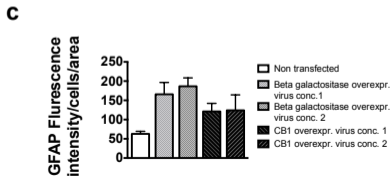

Supplement: Supplementary file 3 — Unchanged GFAP fluorescence intensity after adenoviral overexpression of calcineurin subunit B1 (CB1). (a–c) Primary glia cultures were treated for 72 h with a control adenovirus overexpressing beta-galactosidase (Beta gal) or an adenovirus overexpressing CB1. Overexpression of CB1 increased CB1 protein levels (a) and mRNA levels (b), but had no effect on total GFAP fluorescence intensities compared to beta gal overexpressing cells (c). Beta gal: conc. 1: 38 μl/24-well, conc. 2: 75 μl/24-well; CB1: conc. 1: 75 μl/24-well, conc. 2: 150 μl/24-well. 1 (a, b) and 4–5 technical replicates from glia cells isolated from a pool of four male pups; means ± SEM (c). (PDF 147 kb) [file 12974_2018_1076_MOESM3_ESM.pdf]
